# Development and Evaluation of Individualized Music Therapy for Common Mental Disorders: Protocol for a Multistage Study

**DOI:** 10.2196/90617

**Published:** 2026-07-28

**Authors:** Chunfeng Xiao, Jing Wei, Tao Li, Jinya Cao, Qiaoyan Li, Yanping Duan, Wenqi Geng, Boheng Zhu, Bingzhou Liu, Yongxue Li

**Affiliations:** 1 Peking Union Medical College Hospital, Chinese Academy of Medical Sciences & Peking Union Medical College, 1 Shuaifuyuan, Dongcheng District, Beijing, Beijing, 100730, China, +86-(010)-69151490

**Keywords:** mental disorders, music therapy, treatment, artificial intelligence, mechanism

## Abstract

**Background:**

Pharmacotherapy for common mental disorders is frequently limited by adverse events and suboptimal adherence. While music therapy offers a promising nonpharmacological alternative, its clinical utility is currently constrained by limited accessibility, inconsistent efficacy, and a lack of mechanistic clarity.

**Objective:**

This study aims to describe the development of individualized (receptive) music therapy (IMT), an artificial intelligence (AI)–enabled, neuroscience-guided intervention, and to evaluate its efficacy, safety, and underlying neurobiological mechanisms in adults with major depressive disorder, generalized anxiety disorder, and primary insomnia.

**Methods:**

This multistage research program is being conducted at Peking Union Medical College Hospital and comprises four sequential studies: (1) a cross-sectional pilot study (n=20) to benchmark clinical and electroencephalography features, (2) a prospective cohort study (n=80) evaluating the efficacy and safety of nonindividualized receptive music therapy, (3) a pilot randomized clinical trial (n=300) comparing nonindividualized therapy with IMT over 8 weeks, and (4) a prospective validation study (n=60) of a treatment-response prediction model. Participants include adults aged 18 to 60 years with mild-to-moderate major depressive disorder, generalized anxiety disorder, or primary insomnia, along with healthy controls. In the nonindividualized arm, participants engage in daily 30-minute listening sessions using therapist-curated instrumental tracks designed to regulate mood. In the IMT arm, an AI generation pipeline creates bespoke instrumental tracks based on weekly participant preferences regarding tempo, instrumentation, and emotional valence. To equalize participant–researcher contact across arms and reduce attention and expectation bias, participants in the nonindividualized arm complete a weekly music-experience questionnaire matched in length and timing to the IMT arm’s weekly preference assessment, and treatment credibility and outcome expectancy are measured at baseline in both arms. The primary outcomes are the response rate at 8 weeks (defined as ≥50% reduction in Montgomery-Åsberg Depression Rating Scale [MADRS], Hamilton Anxiety Rating Scale [HAMA], or Pittsburgh Sleep Quality Index [PSQI] scores) and changes in quantitative electroencephalography characteristics. Secondary outcomes include changes in MADRS, HAMA, and PSQI scores from baseline to 8 weeks.

**Results:**

Ethical approval was obtained from the Ethics Review Committee of Peking Union Medical College Hospital (I-24PJ0689). Written informed consent will be obtained from all participants. The recruitment started on May 24, 2024, and the study is expected to be completed by December 2027. Results will be disseminated via peer-reviewed publications, conference presentations, and stakeholder communications. Authorship will follow the International Committee of Medical Journal Editors (ICMJE) criteria. The participant-level dataset will be available upon reasonable request.

**Conclusions:**

This protocol outlines a translational framework designed to address the “therapeutic ceiling” of traditional music therapy. By integrating generative AI with neurophysiological monitoring, this program aims to develop a scalable, precision-medicine approach to mental health care that is both clinically effective and biologically grounded.

## Introduction

Pharmacotherapy remains a cornerstone treatment modality for common mental disorders; however, its long-term efficacy is frequently limited by adverse effects and suboptimal patient adherence [1-3]. Consequently, nonpharmacological interventions have emerged as valuable complementary or alternative therapeutic strategies [4].

Research into music therapy has a long history, during which a coherent theoretical framework and reproducible clinical techniques have gradually evolved [5]. In a seminal definition, Bruscia [6] described music therapy as “a systematic process of intervention wherein the therapist helps the client to promote health, using music experiences and the relationships that develop through them as dynamic forces of change.” As the field has matured, music therapy has expanded beyond receptive listening to encompass recreative and improvisational modalities—active approaches that emphasize participation and creativity. Parallel to this, classification by delivery format has distinguished between individual and group-based care. To date, numerous studies have validated the efficacy and safety of music therapy in improving conditions such as depression [7], anxiety [8], insomnia [9], substance use disorders [10], autism spectrum disorder [11], schizophrenia, and schizophrenia-like disorders [12]. Music therapy has also been shown to enhance cognitive function in patients with Alzheimer disease [13], improve psychological and physical outcomes in patients with cancer [14], and provide comfort in end-of-life care [15].

Investigation into the biological mechanisms of music therapy has identified multiple converging pathways [16]. Music can directly modulate anxiety and stress while functioning as an attentional distractor that reduces ruminative thoughts, thereby promoting positive affect and potentially lowering the risk of relapse associated with negative mood states [17,18]. Neuroendocrine and autonomic effects are also well documented, including reductions in cortisol and favorable changes in heart rate and blood pressure [19-21]. These findings suggest downregulation of hypothalamic-pituitary-adrenal axis activity and restoration of autonomic balance. In patients with insomnia, therapeutic benefits are likely mediated by expectancy effects and classical conditioning, which involve forming an association between presleep music and sleep onset [22,23]. Developmental research on caregiver–infant interaction indicates that early human interaction is imbued with musical features (eg, contour, rhythm, and prosody), providing an ethological foundation for music’s regulatory functions [24]. Furthermore, reward-related mechanisms are implicated. Uplifting music facilitates dopaminergic transmission and modulates limbic circuitry involved in pain and emotion [25-27]. More recently, neuroimaging studies have further elucidated these mechanisms. Resting-state and task-based functional magnetic resonance imaging (MRI) studies have demonstrated that music listening engages widespread cortical and subcortical networks, including the prefrontal cortex, insula, and striatum, with activation patterns that overlap substantially with circuits implicated in mood regulation and reward processing [28,29]. Furthermore, a recent meta-analysis has confirmed that music interventions produce measurable changes in neural connectivity patterns associated with emotional processing [30]. From a clinical psychology perspective, therapeutic change is driven by cognitive, affective, and behavioral factors [31]. Cognitive elements include insight and universalization, while affective elements such as acceptance and altruism contribute to emotional healing. Additionally, prior research has highlighted the mediating role of psychological processes, such as immature defense mechanisms, in the relationship between negative life events and mental disorders [32]. Drawing on psychoanalytic theory, sublimation enables the channeling of emotions through constructive outlets such as art and music [33]. Therefore, music therapy holds the promise of transforming maladaptive defense mechanisms into adaptive ones, offering a unique pathway for psychological healing.

Despite these advances, important limitations of music therapy are increasingly recognized. These notably include restricted access and a “therapeutic ceiling,” whereby conventional music therapy approaches yield moderate but plateauing clinical improvements, beyond which further gains are difficult to achieve using existing methods. Certain active modalities require specialized environments and personnel. Consequently, their availability in routine clinical settings remains limited, and associated costs further constrain uptake. Moreover, effectiveness varies across reports. While recent studies suggest benefits for anxiety and depressive symptoms, comparisons with psychological treatments have shown no significant difference in patient-reported outcomes [7]. Similarly, for insomnia, subjective improvements in sleep quality are often reported, but objective metrics such as total sleep time have demonstrated no significant change in some short-term trials [9].

To address these dual challenges of accessibility and efficacy, we propose a novel approach guided by prior literature and clinical experience: individualized (receptive) music therapy (IMT). This protocol outlines the development of IMT, an artificial intelligence–enabled and neuroscience-guided intervention designed to tailor musical stimuli to patient-specific preferences. We aim to enhance the precision and durability of therapeutic effects in mental health care and to evaluate the efficacy, safety, and mechanisms of action of IMT across multiple stages.

## Methods

### Study Design

**Figure 1.**
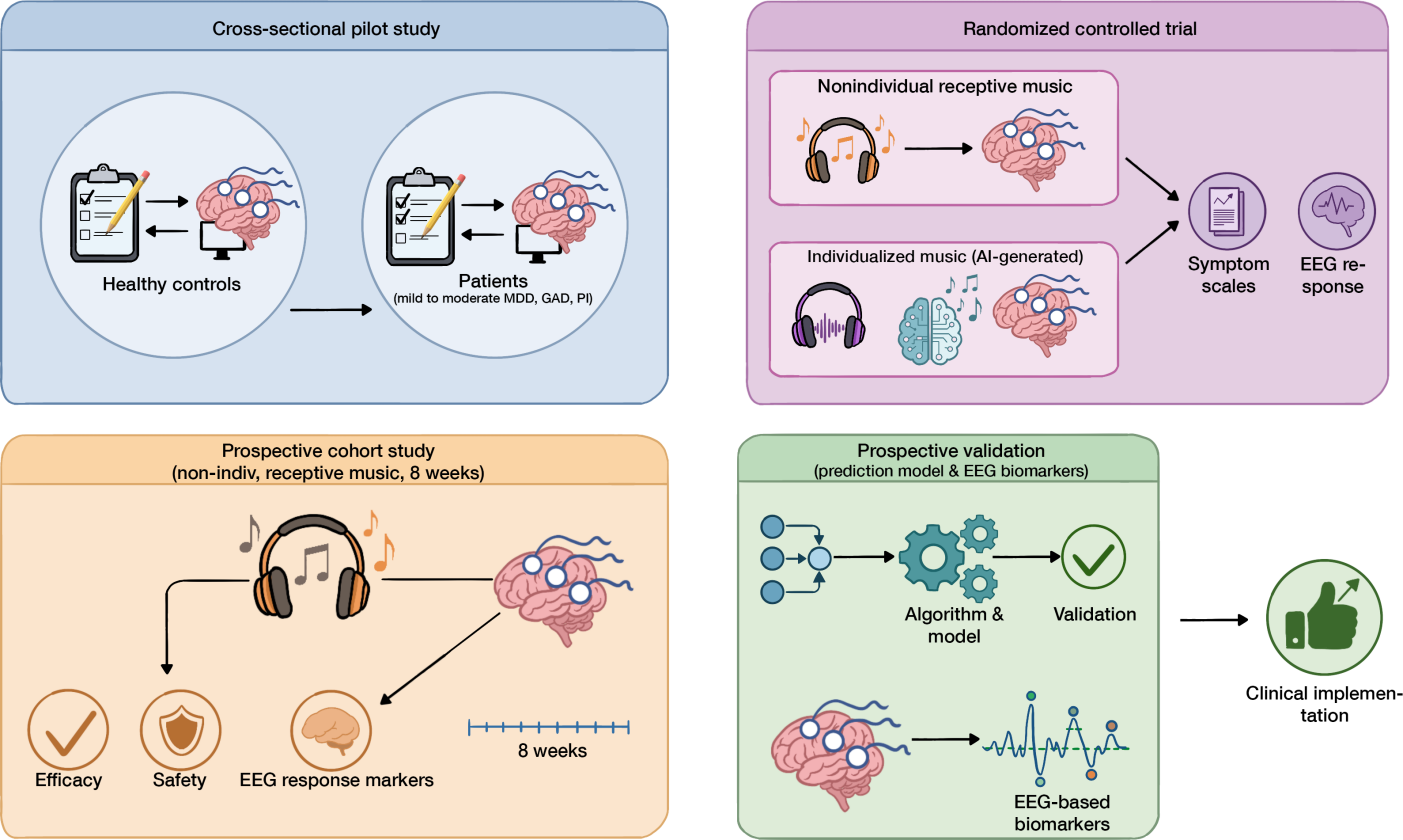
Study flowchart. MDD: major depressive disorder; GAD: generalized anxiety disorder; PI: primary insomnia; non-indiv: nonindividualized; EEG: electroencephalography.

The program comprises 4 sequential studies (Table 1 and Figure 1): 1 cross-sectional study, 2 prospective cohort studies, and 1 randomized controlled trial (RCT). The target populations are adults with mild-to-moderate major depressive disorder (MDD), generalized anxiety disorder (GAD), or primary insomnia (PI), as well as healthy controls. All studies use a common assessment framework including baseline demographics, clinical characteristics, symptom severity, and electroencephalography (EEG), with follow-up assessments at weeks 1, 2, 4, 6, and 8.

**Table 1.** Study description of the individualized (receptive) music therapy research program.

Study	Description	Translational role
Study 1 (Cross-sectional)	A pilot study (n=20; 5 per group: MDD^a^, GAD^b^, PI^c^, and HC^d^) to characterize clinical features and EEG^e^ patterns across the target disorders and healthy controls. These findings will be benchmarked against prior literature to calibrate study procedures.	Findings will be used to calibrate assessment procedures, verify the feasibility of EEG protocols, and generate preliminary hypotheses regarding disorder-specific neurophysiological signatures to guide the analytical framework of subsequent studies.
Study 2 (Prospective Cohort)	An open-label evaluation (n=80; 20 per group: MDD, GAD, PI, and HC) of the efficacy and safety of 8-week nonindividualized receptive music therapy with concurrent EEG monitoring.	Establishes preliminary efficacy benchmarks and identifies EEG features associated with treatment response. These data serve as the reference standard against which the added benefit of individualization is evaluated in study 3.
Study 3 (Randomized Controlled Trial)	A pilot RCT^f^ (n=300; MDD, GAD, and PI) in which participants are randomized 1:1 to receive either nonindividualized receptive music therapy or IMT^g^ for 8 weeks.	Provides the first direct comparison of individualized versus nonindividualized approaches. Clinical and EEG data from studies 1‐3 are pooled to develop a treatment-response prediction model.
Study 4 (Prediction Validation)	A prospective cohort study (n=60) in which all participants receive IMT, and the prespecified prediction model derived from studies 1‐3 is applied at baseline to classify participants as predicted responders or nonresponders.	Validates the prediction model in an independent sample. Unlike study 2, which evaluates the nonindividualized intervention, study 4 exclusively administers IMT and focuses on predictive accuracy rather than treatment efficacy.

a MDD: major depressive disorder.

b GAD: generalized anxiety disorder.

c PI: primary insomnia.

d HC: healthy control.

e EEG: electroencephalography.

f RCT: randomized clinical trial.

g IMT: individualized (receptive) music therapy.

The 4 studies are designed as a sequential translational pipeline in which each stage builds upon the preceding one. Study 1 serves as an exploratory pilot to calibrate clinical and EEG assessment procedures, verify protocol feasibility, and generate preliminary hypotheses regarding disorder-specific neurophysiological features. Although the sample size (n=20) is insufficient for definitive biomarker identification, this study provides essential groundwork for refining the measurement framework used in subsequent stages. Study 2 then evaluates the efficacy and safety of nonindividualized receptive music therapy in a larger cohort, establishing the clinical and neurophysiological benchmarks against which the individualized intervention will be compared. Study 3 directly compares the 2 approaches in a randomized controlled framework, enabling assessment of the incremental benefit of AI-enabled personalization. Finally, study 4 validates a treatment-response prediction model developed from the cumulative data of studies 1 through 3; this study is distinct from study 2 in that all participants receive IMT and the primary objective is to evaluate predictive accuracy rather than treatment efficacy per se.

### Recruitment

Participants are recruited at Peking Union Medical College Hospital via clinician referral and community outreach. Inclusion criteria for the MDD, GAD, and PI groups are as follows: (1) age 18 to 60 years; (2) diagnosis of the relevant disorder according to the *Diagnostic and Statistical Manual of Mental Disorders, Fifth Edition* (*DSM-5*) criteria; (3) severity scores within prespecified ranges (MDD: Hamilton Depression Rating Scale 17-item version [HAMD-17] score>7 and ≤23; GAD: HAMA score>7 and ≤21) or, for PI, mild-to-moderate severity with predominant sleep-onset difficulty persisting longer than 3 months; and (4) capacity to provide written informed consent. Exclusion criteria include the following: (1) any current or lifetime history of comorbid mental disorders, such as bipolar disorder, obsessive-compulsive disorder, or substance use disorder; (2) severe or unstable medical conditions; (3) pregnancy or lactation; and (4) sensory or cognitive impairments, such as hearing loss or intellectual disability, that would preclude participation.

The use of the HAMD-17 for screening and the MADRS for outcome assessment reflects the complementary psychometric properties of these instruments. The HAMD-17 is widely used as a screening and severity-grading instrument in clinical practice and provides well-established severity thresholds for defining mild-to-moderate depression. The MADRS, by contrast, offers superior sensitivity to change over time and is recommended as a primary outcome measure in clinical trials owing to its focus on core depressive symptoms and reduced somatic item content, which minimizes confounding by physical comorbidities [34].

### Concomitant Treatments

During the study period, the use of any psychotropic medications or other treatments for the target disorder is prohibited in principle, including psychotherapy and neuromodulation therapies (eg, transcranial magnetic stimulation). Medications for the management of treatment-emergent adverse events are permitted. Any concomitant medications or treatments used during the study, including those administered inadvertently, are documented in the case report forms, with details of drug name, dosage, dates of administration, and indication recorded at each visit. Participants who require initiation of prohibited treatments owing to clinical deterioration will be withdrawn from the study and will receive appropriate clinical care.

### Sample Size Calculation

Study 3 is a pilot RCT designed to assess feasibility and to generate preliminary effect estimates to inform a definitive future trial. Accordingly, the sample size is based on precision estimation rather than formal power calculation. The primary outcome is the response rate at 8 weeks, defined as a reduction of 50% or greater from baseline in the disorder-specific symptom severity score. We adopted a minimal clinically important difference–anchored CI approach, setting a 20-percentage-point absolute difference in response rates as the threshold for clinical importance. With 50 participants per arm within each diagnostic group, the expected 1-sided 80% CI half-width for the between-group difference in response rates is approximately 8 to 12 percentage points (derived from a response rate of 70% for the control group and a range of between-group differences from 0% to 30%), thereby permitting precise estimation of feasibility metrics and preliminary efficacy signals to plan the definitive trial.

### Randomization and Blinding

For the RCT, an independent biostatistician will generate the allocation sequence using SAS software (version 9.4; SAS Institute). Randomization will be managed via a secure, web-based system to ensure allocation concealment. Upon confirmation of eligibility and completion of baseline assessments, the system will release the assignment to a research nurse who is not involved in outcome assessment. Outcome assessors will remain blinded to group assignment throughout the trial. Given the nature of the intervention, complete participant blinding is not feasible, as those in the IMT arm engage in weekly preference assessments and music selection, whereas participants in the nonindividualized arm receive therapist-curated tracks without weekly customization. Beyond the impossibility of participant blinding, we explicitly recognize that the weekly preference assessment and track-rating procedure in the IMT arm constitutes an additional, potentially active, component of participant–researcher interaction and engagement that is not present in a conventional nonindividualized protocol. Any between-arm difference in outcomes could therefore reflect not only the personalization of the musical stimulus itself but also this greater attention, engagement, and sense of agency. To mitigate both expectation bias and this attention and engagement confound, several strategies are used: (1) participants in both arms are informed only that they will receive music therapy, without being told which approach is hypothesized to be superior; (2) all participants complete equivalent contact time and daily listening schedules; specifically, participants in the nonindividualized arm complete a weekly music-experience questionnaire covering their listening experience, mood, and engagement, that is matched to the IMT arm’s weekly preference assessment in length, frequency, mode of administration, and degree of staff contact, so that the 2 arms are equated for attention and procedural engagement and differ only in whether the questionnaire responses are used to regenerate the following week’s music; and (3) primary outcomes are assessed by independent raters who have no knowledge of treatment allocation. In addition, to render expectation effects measurable rather than merely assumed, all participants complete a validated treatment-credibility and outcome-expectancy measure (the Credibility/Expectancy Questionnaire, CEQ) after allocation and before the first session; baseline expectancy scores will be compared between arms and entered as a covariate in prespecified sensitivity analyses of the primary outcomes. To assess the integrity of assessor blinding and to characterize residual participant unblinding, both participants and outcome assessors will be asked to guess the participant’s allocation at week 8, and a blinding index (bang blinding index) will be computed and reported. Unblinding of assessors is permitted only in the event of a medical emergency.

### Interventions

#### Nonindividualized Receptive Music Therapy

**Figure 2.**
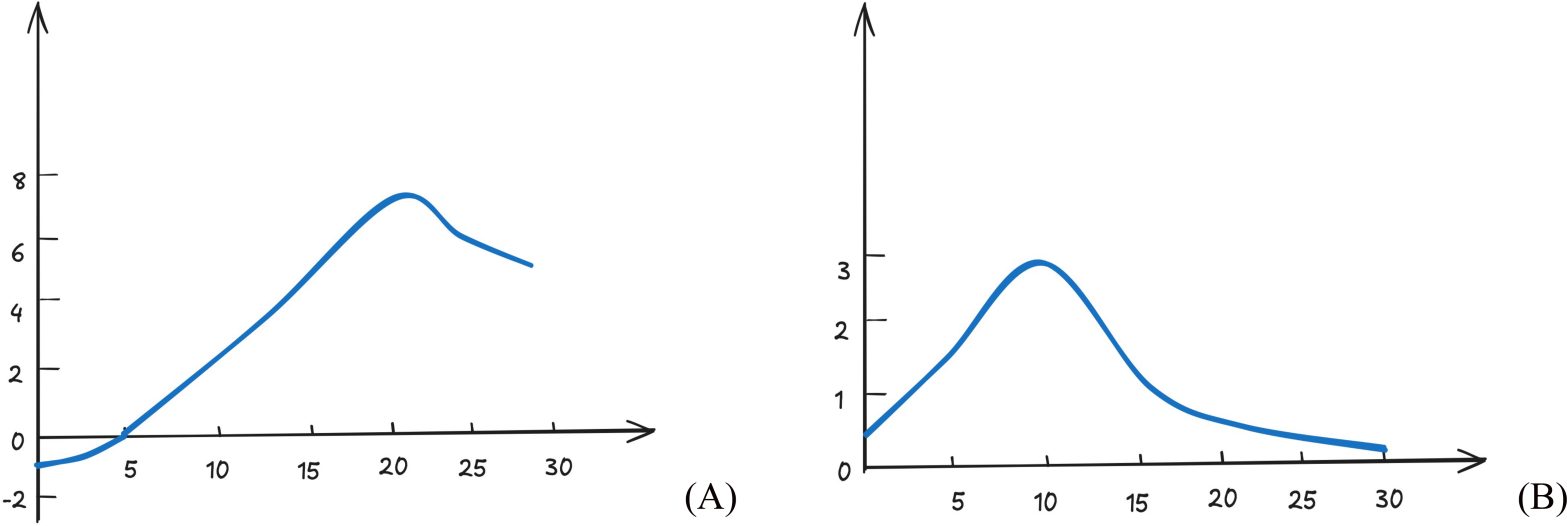
Music affective trajectories used in nonindividualized receptive music therapy (**A**) Affective trajectory for participants with major depressive disorder or generalized anxiety disorder, showing a gradual upward shift in target emotional valence (low → higher → moderate and settled) across the 30-minute session. The x-axis represents session time (minutes 0‐30); the y-axis represents target arousal and valence level on a standardized scale (higher score indicates higher arousal and positive valence). (**B**) Biphasic affective trajectory for participants with primary insomnia (PI) showing a biphasic trajectory. The x-axis represents session time (minutes 0‐30); the y-axis represents target arousal level (higher score indicates higher arousal and positive valence). The trajectory features a gentle initial rise in affect to reduce negative cognitions, followed by a progressive taper (slower tempo, simplified rhythm and melody, increased repetition) to lower physiological arousal before sleep.

Participants engage in daily 30-minute instrumental listening sessions curated by a certified music therapist. The music therapists hold a master’s degree or higher in music therapy with a minimum of 5 years of clinical experience in psychiatric settings. Track selection follows a standardized protocol based on the predefined affective trajectory framework (Figure 2), and the final track list for each participant is reviewed by a second member of the research team to verify adherence to the selection criteria. Selection adheres to the “ISO principle” of mood entrainment [35]. Music initially matches the participant’s current affect and gradually shifts toward an adaptive target state. Before treatment, participants complete the Short Test of Music Preferences-Revised (STOMP-R), which assesses liking for 23 musical styles grouped into 4 categories on a 1‐7 scale (1=dislike strongly; 7=like strongly) [36,37]. Based on prior site-specific data showing high preference for “Reflective & Complex” and “Upbeat & Conventional” styles, the therapist selects instrumental tracks while excluding lyrical content to minimize semantic processing. Each session comprises 6 to 10 excerpts ordered to follow a specific affective trajectory: (1) for MDD and GAD: The sequence guides affect upward (eg, calm and sad → warm → cheerful → relaxed; Figure 2A); (2) for PI: The sequence follows a biphasic trajectory. It initially elevates affect to reduce negative rumination, then progressively decreases tempo and complexity to facilitate sleep onset (Figure 2B).

In addition to the listening intervention, participants in the nonindividualized arm complete a brief weekly music-experience questionnaire that mirrors the contact time and structure of the weekly preference assessment used in the IMT arm. This questionnaire records the participant’s listening experience, mood, and engagement but is not used to alter the curated track list; its purpose is to equate the 2 arms for participant–researcher contact and procedural engagement, thereby reducing differential attention and expectation effects.

#### IMT

IMT uses an artificial intelligence (AI)–enabled pipeline to tailor the interventions to weekly patient preferences. Before each week’s session, participants complete a preference questionnaire across 6 dimensions: emotion, style, intensity, instrumentation, variation, and tempo. These inputs are converted into standardized prompts for the Suno music platform (V4 model; API: sunoapi.org). For example, a selection of “calm, Western Classical, flute and piano and cello, slow tempo” is programmatically rendered into a specific generation prompt. The system generates 16 unique instrumental tracks, which the participant rates on a 1‐7 scale. The 8 highest-rated tracks are compiled into the 30-minute intervention set for that week. To ensure therapeutic consistency across participants, the AI-generated tracks are subject to predefined acoustic constraints. All generated tracks are required to be purely instrumental (lyrical content is excluded). Furthermore, key acoustic features, including tempo (beats per minute), spectral centroid, and rhythmic complexity, are logged for each generated track. The therapeutic structure of the IMT condition mirrors that of the nonindividualized arm in session duration (30 min daily), number of tracks per session (6 to 10 excerpts), and overall affective trajectory framework. The sole difference lies in the source of the musical material (AI-generated based on individual preferences vs therapist-curated from existing libraries). Participants are instructed to listen once daily in a quiet environment and to focus their attention on the auditory experience and the emotions it evokes. The playback interface displays the following guidance: “Please adjust your posture to feel relaxed and comfortable. Close your eyes and begin deep breathing. After the music begins, focus all your attention on the music and the feelings it evokes.”

### Intervention Fidelity and Adherence Monitoring

Several measures are implemented to ensure intervention fidelity and to monitor participant adherence. Daily listening compliance is tracked automatically through the custom-developed mobile app, which records session initiation time, duration of playback, and completion status for each listening session. Adequate adherence is defined a priori as completion of at least 80% of scheduled listening sessions (ie, a minimum of 45 of 56 daily sessions over the 8-week intervention period). Participants falling below this threshold will be classified as nonadherent for the purpose of per-protocol analysis, although all randomized participants will be included in the primary intention-to-treat analysis regardless of adherence level. To ensure the quality and consistency of AI-generated music in the IMT arm, a 2-stage quality assurance process is used. First, all generated tracks are screened against predefined acoustic parameters (as described above) to exclude those falling outside acceptable ranges. Second, a certified music therapist reviews a random sample of generated tracks each week (approximately 20% of total output) to verify that the musical content is therapeutically appropriate, free of unexpected artifacts, and consistent with the intended affective trajectory. Any quality concerns are flagged and addressed before participant delivery. For the nonindividualized arm, intervention fidelity is maintained using a standardized track library curated by the same certified music therapist, with track selection following the predefined affective trajectory protocols. Session delivery is identical across both arms in terms of duration, frequency, and environmental instructions.

### Study Procedure and Data Collection

Recruitment occurs at Peking Union Medical College Hospital through clinician referrals and community outreach. Following screening and provision of written informed consent, participants are assigned a unique alphanumeric study code (eg, IMT-1‐001). Eligible participants receive training on a custom-developed mobile app, which serves as the platform for collecting patient-reported outcomes and for delivering the daily musical intervention. Data acquisition encompasses six primary domains:

1. Demographics and history: age, sex, education, and detailed medical and psychiatric history
2. Symptom severity: assessed using the Montgomery-Åsberg Depression Rating Scale (MADRS), Hamilton Anxiety Rating Scale (HAMA), Pittsburgh Sleep Quality Index (PSQI), Clinical Global Impression–Severity (CGI-S), and Immediate Mood Scale (IMS)
3. Psychosocial functioning: evaluated via the Unified Psychophysiological-Psychosocial Assessment Questionnaire (UPPSAQ-70), Defense Style Questionnaire (DSQ), and Toronto Alexithymia Scale (TAS), etc
4. Neurophysiology: includes optional assessments such as electroencephalography (EEG), functional near-infrared spectroscopy (fNIRS), MRI, and heart rate variability (HRV)
5. Treatment credibility and expectancy: assessed at baseline (after allocation, before the first session) in studies 2 and 3 using the CEQ to permit measurement and statistical adjustment for expectation effects
6. Safety: monitored using the Young Mania Rating Scale (YMRS), the Brief Psychiatric Rating Scale (BPRS-4) positive symptom subscale, the UKU Side Effect Rating Scale, the Frequency, Intensity, and Burden of Side Effects Rating (FIBSER), and the Columbia-Suicide Severity Rating Scale (C-SSRS)

Follow-up visits occur at weeks 1, 2, 4, 6, and 8. These assessments focus on symptom severity, neurophysiology, and safety. At baseline in studies 2 and 3, participants additionally complete the Short Test of Music Preferences-Revised (STOMP-R). In study 4, baseline procedures include the application of the treatment-response prediction model derived from earlier datasets. For participants undergoing EEG, protocols include resting-state, P300, and mismatch negativity (MMN) paradigms. Additionally, a music-stimulation task (approximately 20 min of curated excerpts) is conducted at baseline and week 8 to characterize neural reactivity to the intervention.

### Treatment-Response Prediction Model

The treatment-response prediction model to be validated in study 4 will be developed using data from studies 1 through 3. Candidate predictors will include baseline demographic characteristics (age, sex, and education), clinical variables (baseline symptom severity scores, disorder diagnosis, duration of illness, and medication status), music preference profiles (STOMP-R scores), and baseline neurophysiological features (resting-state EEG spectral power and frontal alpha asymmetry). The outcome variable for model development will be treatment response at 8 weeks, defined as a reduction of 50% or greater in the disorder-specific primary outcome measure. Model development will use logistic regression with regularization (elastic net) to handle the potentially large number of candidate predictors relative to the sample size, with internal validation using 10-fold cross-validation. The final model will be prespecified and locked before its application to the study 4 validation cohort. Model performance in study 4 will be assessed using discrimination metrics (area under the receiver operating characteristic curve, sensitivity, and specificity) and calibration measures (Hosmer–Lemeshow test and calibration plots). Should the cumulative sample from studies 1 through 3 prove insufficient for robust model development, alternative approaches such as penalized regression or decision-tree methods will be considered, and the model validation in study 4 will be framed accordingly. A detailed statistical analysis plan for the prediction model will be finalized before study 4 enrollment commences.

### Outcome Measures

#### Primary Outcome Measure

Response rate at 8 weeks, defined as ≥50% reduction in MADRS, HAMA, or PSQI scores; change in quantitative EEG characteristics from baseline to week 8. Specific EEG features of interest are detailed in the statistical analysis plan.

#### Secondary Outcome Measure

The secondary outcome is change in MADRS, HAMA, and PSQI scores.

#### Safety Outcome Measure

General side effects were measured by the UKU and FIBSER. Psychotogenic effects were measured with the BPRS consisting of suspiciousness, hallucinations, unusual thought content, and conceptual disorganization. Manic symptoms were assessed using YMRS. C-SSRS was also rated throughout the trial for safety purposes.

### Plan for Statistical Analysis

All statistical analyses will be performed using SPSS Statistics (version 23; IBM Corp), R (version 4.3 or higher; R Foundation for Statistical Computing), and MATLAB (MathWorks) with the EEGLAB toolbox for EEG data processing. All hypothesis tests will be 2-sided, with a significance level of *α*=.05.

#### Descriptive and Baseline Analyses

Continuous variables with approximately normal distributions will be summarized as mean (SD) and compared between groups using independent-samples 2-tailed *t* tests (with Welch correction when variances are unequal); nonnormally distributed continuous variables will be summarized as median (IQR) and compared using the Mann-Whitney *U* test. Categorical variables will be summarized as counts (%) and compared using chi-square tests (continuity-corrected for 2×2 tables) or the Fisher exact test when expected cell counts are below 5. Baseline demographic and clinical characteristics will be compared across groups to assess the adequacy of randomization in study 3, and baseline CEQ scores will be compared between arms.

#### Primary Outcome Analysis

For the response rate at 8 weeks, between-group comparisons will be conducted using chi-square tests, with results reported as risk difference and risk ratio with corresponding 95% CIs. To accommodate the repeated-measures structure of the data (assessments at baseline and at weeks 1, 2, 4, 6, and 8), linear mixed-effects models for repeated measures (MMRM) will be used for continuous outcomes, with fixed effects for treatment group, time, and their interaction; baseline scores will be included as covariates; and random intercepts for participants will be specified to accommodate within-subject correlation. Because study 3 cannot blind participants and the IMT arm involves additional engagement, 2 prespecified sensitivity analyses will probe the robustness of the primary treatment effect to expectation and attention effects: (1) the MMRM and the response-rate models will be refitted with baseline CEQ scores added as covariates; and (2) the analyses will be repeated with adjustment for the Bang Blinding Index to explore whether the estimated treatment effect is attenuated after accounting for residual unblinding. Concordance between the primary (unadjusted) and these expectancy-adjusted estimates will be interpreted as evidence regarding the extent to which any observed benefit is attributable to personalization of the stimulus rather than to differential expectancy or attention. Time to response or remission will be compared between groups using Kaplan–Meier survival curves and the log-rank test. For study 3, the primary analysis will follow an intention-to-treat principle, including all randomized participants according to their assigned group. A supplementary per-protocol analysis will include only participants who complete at least 80% of scheduled listening sessions (ie, a minimum of 45 of 56 daily sessions).

#### Missing Data

Missing data patterns will be examined and characterized as missing completely at random (MCAR), missing at random (MAR), or missing not at random (MNAR) using Little’s MCAR test and pattern analyses. Under the MAR assumption, multiple imputation by chained equations (MICE) will be used to generate 20 imputed datasets, and results will be pooled using Rubin’s rules. As a sensitivity analysis, last-observation-carried-forward (LOCF) imputation will be applied within the repeated-measures ANOVA framework, in line with our prespecified protocol. Additional sensitivity analyses under MNAR scenarios, including tipping-point analyses, will be conducted to evaluate the robustness of the findings.

#### Heterogeneity of Target Disorders, Subgroup Analyses, and Multiple Comparisons

Recognizing that MDD, GAD, and PI differ in symptom profiles and neurobiological mechanisms, the primary analysis of study 3 will pool all 3 disorders to maximize statistical power, with diagnostic group included as a stratification factor in the randomization and as a covariate in all pooled models. Treatment × diagnosis interaction terms will be included in the mixed-effects models to assess whether treatment effects vary by disorder. If significant heterogeneity is detected, disorder-specific results will be reported separately. The analytic hierarchy is specified a priori as follows. The pooled treatment effect (IMT vs nonindividualized therapy), adjusted for diagnosis, is the confirmatory primary contrast. Disorder-specific treatment effects (MDD, GAD, and PI analyzed separately) are prespecified secondary, hypothesis-generating analyses. For each disorder, we will report the response-rate difference and the MMRM-derived between-group difference in symptom change with 95% confidence intervals, regardless of statistical significance, so that the direction and precision of the per-disorder estimates are transparent and can inform the design of a future definitive trial. The treatment × diagnosis interaction will be evaluated primarily as a test of effect-modification rather than as a gatekeeper for reporting; disorder-specific estimates will be presented whether or not the interaction term reaches significance, but will be explicitly labeled exploratory. We acknowledge that this pilot is not powered for confirmatory subgroup inference. With approximately 50 participants per arm per disorder (100 per diagnostic group), each disorder-specific contrast has limited power to detect between-arm differences smaller than a large effect, and the trial is powered for precision of estimation rather than for hypothesis testing within subgroups (see Sample Size Calculation). To avoid over-interpreting unstable subgroup estimates, the disorder-specific analyses will be supplemented by a hierarchical Bayesian model in which disorder-specific treatment effects are treated as exchangeable and shrunk toward the overall pooled effect; this borrows strength across the 3 disorders and yields more stable, less extreme per-disorder estimates than fully separate analyses. Posterior means and 95% credible intervals for each disorder will be reported alongside the frequentist estimates. We will not draw definitive efficacy conclusions for any individual disorder from study 3; rather, the per-disorder estimates will be used to generate hypotheses and to inform stratified sample-size planning for the definitive trial. It should be noted that the music therapy protocol uses the same affective trajectory for participants with MDD and GAD, whereas a distinct biphasic trajectory is used for participants with PI. This difference in trajectory design reflects the distinct clinical objectives for each disorder and will be accounted for in the analysis by including trajectory type as a covariate in sensitivity analyses. Because trajectory type is collinear with the PI-versus-(MDD and GAD) distinction, this covariate adjustment will be reported as a sensitivity analysis complementary to the disorder-stratified analyses above. For the family of disorder-specific secondary contrasts, the false discovery rate will be controlled using the Benjamini–Hochberg procedure, and both adjusted and unadjusted *P* values will be reported.

#### EEG Data Analysis

EEG data will be preprocessed using the EEGLAB toolbox (version 2024.0 or later) implemented in MATLAB. The preprocessing pipeline will include: (1) bandpass filtering (0.5‐45 Hz); (2) downsampling to 250 Hz; (3) detection of bad channels followed by spherical-spline interpolation; (4) rejection of bad segments exceeding ±100 μV using a sliding-window approach; (5) re-referencing to the average reference; (6) artifact rejection using independent component analysis (ICA) with automated classification (eg, ICLabel) to remove ocular, myogenic, and cardiac artifacts; and (7) epoching and baseline correction. Quantitative EEG features of interest include resting-state spectral power across the delta (1‐4 Hz), theta (4‐8 Hz), alpha (8‐13 Hz), beta (13‐30 Hz), and gamma (30‐45 Hz) bands; frontal alpha asymmetry; and event-related potential (ERP) measures, including P300 amplitude and latency and MMN amplitude and latency. For the music-stimulation task, changes in spectral power and functional connectivity (measured by phase-locking value) between baseline and week 8 will be examined. The EEG analysis is structured to respect both the multiplicity of neural metrics and the heterogeneity of the 3 disorders. EEG outcomes will be analyzed using mixed-effects models with fixed effects for treatment group, time, diagnostic group, and their interactions, and random intercepts for participants; this allows disorder-specific neurophysiological responses to be estimated within a single model while accounting for the repeated-measures structure. To control the false discovery rate without diluting power across unrelated metrics, the Benjamini–Hochberg procedure will be applied within prespecified feature families (resting-state spectral power across bands; frontal alpha asymmetry; ERP components; and task-related connectivity) rather than as a single global correction across all EEG metrics. For the spatially and spectrally resolved resting-state and connectivity analyses, cluster-based permutation testing will additionally be used to control family-wise error while accounting for correlations across adjacent electrodes, frequencies, and time points. EEG outcomes are designated as co-primary for mechanistic exploration should sample sizes prove insufficient for robust neurophysiological inference. However, we explicitly acknowledge that, with 20 participants per group in studies 1‐2 and disorder-specific subsamples in study 3, the neurophysiological analyses are not powered for confirmatory inference. Accordingly, EEG findings will be interpreted as exploratory and mechanistic, prioritizing estimation (effect sizes with confidence or credible intervals) over null-hypothesis significance testing, and disorder-specific EEG effects will be reported descriptively to generate hypotheses for the definitive trial.

#### Assumption Checks and Robustness

Assumptions for parametric analyses (normality and homogeneity of variance) will be assessed using the Shapiro–Wilk test, Q–Q plots, and the Levene test. When assumptions are not met, nonparametric or variance-robust alternatives will be applied. All analyses will adhere to a prespecified statistical analysis plan that will be finalized before database lock.

### Data Management

Clinical and demographic data are recorded on standardized case report forms (CRFs) and tracked via study identification codes. To ensure data integrity, a designated investigator conducts weekly quality assurance reviews to verify completeness and internal consistency. Signed informed consent documents are stored in secure, restricted-access files separate from the CRFs. The final dataset undergoes a comprehensive validation review by a senior investigator prior to statistical analysis.

### Ethical Considerations

The Ethics Review Committee of Peking Union Medical College Hospital, Chinese Academy of Medical Sciences, approved the study (approval number I-24PJ0689), which complies with the Declaration of Helsinki, and was registered on the Chinese Clinical Trial Registry (identifier ChiCTR2400083246; registered April 2024). Before enrollment, investigators will explain potential benefits and risks, and participants will provide written informed consent. Participant confidentiality will be protected throughout the study by restricting data access to authorized investigators. Treatment-emergent adverse events will be managed at no cost to participants at the study institution. Study findings will be presented at scientific conferences and submitted to peer-reviewed journals. Authorship will follow International Committee of Medical Journal Editors (ICMJE) criteria, and the manuscript will be written and revised by the authors; no professional writers will be used. The protocol is registered with the Chinese Clinical Trial Registry, and the participant-level dataset will be available from the corresponding author upon reasonable request.

## Results

The study was funded by the National Natural Science Foundation of China (T2341003) in 2023. Recruitment officially commenced on May 24, 2024. As of submission, the study is ongoing, with data collection expected to be completed by December 2027.

## Discussion

### Principal Findings

This protocol describes a multistage research program designed to develop, evaluate, and optimize an AI-enabled IMT intervention for common mental disorders. We hypothesize that IMT, by tailoring musical stimuli to patient-specific preferences through generative AI, will yield superior response rates compared with nonindividualized receptive music therapy, and that these therapeutic effects will be accompanied by measurable changes in quantitative EEG characteristics. The sequential study design enables iterative refinement: study 1 establishes neurophysiological benchmarks, study 2 provides preliminary efficacy data for the nonindividualized protocol, study 3 directly compares the 2 approaches in an RCT, and study 4 validates a prediction model to identify patients most likely to benefit from IMT.

### Comparison With Prior Work

Our program addresses 2 critical limitations in current music therapy practice: restricted accessibility and a “therapeutic ceiling” on efficacy. We adopted a receptive music therapy framework owing to its high feasibility and simplicity. This approach supports a scalable, clinic-to-home model where patients listen via headphones. To enhance therapeutic potency, we focus on the intervention’s core substrate: the musical stimulus itself. Unlike prior studies that selected from existing commercial libraries, our approach uses generative AI to create customized instrumental tracks. This allows for fine-grained tailoring based on granular participant preferences within a standardized therapeutic structure.

Neuroimaging research indicates that mental disorders are characterized by abnormalities in large-scale network connectivity, particularly within circuits governing affect, reward, and the default mode network [38,39]. Concurrent research demonstrates that music modulates these same networks [40]. For instance, preferred music enhances connectivity within the default mode network and alters coupling between the auditory cortex and the hippocampus, a region central to memory and emotion [41]. We hypothesize that targeted musical stimulation can ameliorate disorder-relevant dysfunction by modulating activity within these specific circuits. While functional overlap exists between brain regions implicated in psychiatric illness and those engaged by music, the therapeutic implications require targeted investigation. Consequently, this protocol adopts a neuroscience-guided approach that moves beyond simple preference-based personalization toward a biologically grounded model of IMT.

Neural activity monitoring via EEG serves as a primary tool for the development and refinement of IMT in this program. These techniques enable the observation of brain responses to musical stimulation and the delineation of biological response patterns. For example, preliminary evidence suggests that melody and rhythm may differentially engage language and motor cortices [42]. Correlating these biological markers with clinical outcomes provides an objective basis for the personalized selection of musical materials. Furthermore, real-time monitoring may eventually enable dynamic adjustment of treatment parameters based on brain-state changes. Consistent with this rationale, EEG is embedded throughout the program to track objective markers and to iteratively strengthen the neuroscientific basis of music therapy.

### Strengths and Limitations

This protocol has several strengths. First, the multistage design provides a structured translational pathway, progressing from mechanistic exploration through preliminary efficacy assessment to randomized comparison and predictive model validation. This sequential approach allows findings from each stage to inform the design and conduct of subsequent studies. Second, the integration of generative AI technology enables a degree of personalization that would be difficult to achieve through conventional music therapy practice, offering a scalable model that can be readily adapted across clinical settings. Third, the incorporation of neurophysiological monitoring alongside clinical outcome measures provides an opportunity to examine the biological mechanisms underlying treatment response, moving beyond purely symptom-based evaluation.

Several limitations should also be noted. First, the open-label design of study 2 precludes causal attribution of observed improvements to the intervention, as natural symptom fluctuation and regression to the mean cannot be excluded in the absence of a control group. However, this limitation is mitigated by the inclusion of a randomized controlled comparison in study 3. Second, complete participant blinding in the RCT is not feasible owing to the inherent differences between the 2 intervention arms, which may introduce differential expectancy effects; moreover, the weekly preference assessment in the IMT arm provides additional participant–researcher contact, engagement, and a sense of agency that is absent from the nonindividualized arm, so that any observed superiority of IMT could in principle reflect this added attention and engagement (a cointervention and attention effect) rather than personalization of the musical stimulus per se. To address these concerns, we have implemented several strategies to minimize bias, as described in the Randomization and Blinding section, including an attention-matched weekly questionnaire in the nonindividualized arm, baseline measurement of treatment credibility and expectancy with expectancy-adjusted sensitivity analyses, and a formal blinding-integrity (bang blinding index) assessment. We nonetheless recognize that these measures reduce, but cannot fully eliminate, the confounding of personalization with attention and expectancy; cleanly separating these components would require designs such as a 3-arm trial incorporating an attention-matched sham-personalization control, which we identify as an important direction for the definitive trial. Third, the pilot RCT is powered for precision of estimation rather than confirmatory hypothesis testing; consequently, the study may be underpowered to detect clinically meaningful differences within individual diagnostic subgroups, and subgroup analyses should be interpreted as exploratory. Because MDD, GAD, and PI differ in symptom profiles, neurobiology, and even in the affective trajectory of the intervention delivered, pooling them maximizes power for the overall comparison but cannot resolve whether the 3 disorders respond differently to IMT; we have therefore prespecified disorder-stratified estimation, a treatment × diagnosis interaction test, and a hierarchical Bayesian model that shrinks per-disorder estimates toward the pooled effect, and we explicitly refrain from drawing disorder-specific efficacy conclusions from this pilot. Definitive, disorder-specific inference will require an adequately powered, stratified trial. Fourth, all studies conclude at 8 weeks, and the durability of treatment effects beyond the intervention period remains unknown. Finally, although EEG offers excellent temporal resolution for capturing dynamic neural responses to music, it provides limited spatial resolution compared with functional MRI, which may constrain the ability to localize treatment-related changes in deep brain structures; in addition, the EEG analyses are exploratory and not powered for confirmatory neurophysiological inference, particularly at the level of individual disorders.

### Future Directions

If the pilot RCT demonstrates promising results, a fully powered, multicenter definitive trial will be warranted to confirm the efficacy of IMT across diverse clinical populations. To disentangle the personalization effect from the additional attention and engagement inherent in the IMT procedure, the definitive trial should consider a 3-arm design that adds an attention-matched sham-personalization control (in which participants complete weekly preference ratings but receive nonindividualized music), enabling the specific contribution of stimulus personalization to be isolated. Future studies should also incorporate longer follow-up periods (eg, 12 to 24 wk) to assess the durability of therapeutic effects and the potential for relapse prevention. Adequately powered, diagnosis-stratified samples will be required to draw firm disorder-specific conclusions for MDD, GAD, and PI, and to support confirmatory neurophysiological inference. The prediction model developed in study 4, if validated, could serve as a clinical decision-support tool for identifying patients most likely to benefit from individualized interventions; further refinement using machine-learning approaches with larger datasets would enhance its generalizability. Additionally, the integration of multimodal neuroimaging (combining EEG with fMRI or fNIRS) in future protocols could provide a more comprehensive understanding of the neural circuits engaged by IMT. Finally, investigating the applicability of the IMT framework to other psychiatric conditions, as well as to pediatric and geriatric populations, represents an important direction for extending the reach of this approach.

### Conclusions

This protocol outlines a translational framework to overcome the “therapeutic ceiling” of traditional music therapy. By integrating generative AI with neurophysiological monitoring, this program aims to establish a scalable, precision-medicine approach to mental health care that is both clinically effective and biologically grounded.

## Supplementary material

10.2196/90617 Checklist 1 SPIRIT checklist.

## Abbreviations

BPRS: Brief Psychiatric Rating Scale
C-SSRS: Columbia-Suicide Severity Rating Scale
CEQ: Credibility/Expectancy Questionnaire
CGI-S: Clinical Global Impression–Severity
CRF: case report form
DSQ: Defense Style Questionnaire
EEG: electroencephalography
ERP: event-related potential
FIBSER: Frequency, Intensity, and Burden of Side Effects Rating
GAD: generalized anxiety disorder
HAMA: Hamilton Anxiety Rating Scale
HAMD-17: Hamilton Depression Rating Scale 17-item version
HRV: heart rate variability
ICMJE: International Committee of Medical Journal Editors
IMS: Immediate Mood Scale
IMT: individualized (receptive) music therapy
MADRS: Montgomery-Åsberg Depression Rating Scale
MAR: missing at random
MCAR: missing completely at random
MDD: major depressive disorder
MICE: multiple imputation by chained equation
MMN: mismatch negativity
MMRM: mixed-effects models for repeated measure
MNAR: missing not at random
MRI: magnetic resonance imaging
PSQI: Pittsburgh Sleep Quality Index
RCT: randomized clinical trial
STOMP-R: Short Test of Music Preferences-Revised
TAS: Toronto Alexithymia Scale
UPPSAQ-70: Unified Psychophysiological-Psychosocial Assessment Questionnaire
YMRS: Young Mania Rating Scale
